# LVGG-IE: A Novel Lightweight VGG-Based Image Encryption Scheme

**DOI:** 10.3390/e26121013

**Published:** 2024-11-23

**Authors:** Mingliang Sun, Jie Yuan, Xiaoyong Li, Dongxiao Liu, Xinghai Wei

**Affiliations:** Key Laboratory of Trustworthy Distributed Computing and Service (BUPT), Ministry of Education, Beijing University of Posts and Telecommunications, Beijing 100876, China; sunml2018@bupt.edu.cn (M.S.); lixiaoyong@bupt.edu.cn (X.L.); liudongxiao@bupt.edu.cn (D.L.); junjuntvt@bupt.edu.cn (X.W.)

**Keywords:** VGG, image encrypt, S-box, single-connected layer, chaos

## Abstract

Image security faces increasing challenges with the widespread application of computer science and artificial intelligence. Although chaotic systems are employed to encrypt images and prevent unauthorized access or tampering, the degradation that occurs during the binarization process in chaotic systems reduces security. The chaos- and DNA-based image encryption schemes increases its complexity, while the integration of deep learning with image encryption is still in its infancy and has several shortcomings. An image encryption scheme with high security and efficiency is required for the protection of the image. To address these problems, we propose a novel image encryption scheme based on the lightweight VGG (LVGG), referred to as LVGG-IE. In this work, we design an LVGG network with fewer layers while maintaining a high capacity for feature capture. This network is used to generate a key seed, which is then employed to transform the plaintext image into part of the initial value of a chaotic system, ensuring that the chaos-based key generator correlates with the plaintext image. A dynamic substitution box (S-box) is also designed and used to scramble the randomly shuffled plaintext image. Additionally, a single-connected (SC) layer is combined with a convolution layer from VGG to encrypt the image, where the SC layer is dynamically constructed by the secret key and the convolution kernel is set to 1×2. The encryption efficiency is simulated, and the security is analyzed. The results show that the correlation coefficient between adjacent pixels in the proposed scheme achieves $10^{-4}$. The NPCR exceeds 0.9958, and the UACI falls within the theoretical value with a significance level of 0.05. The encryption quality, the security of the dynamic S-box and the SC layer, and the efficiency are tested. The result shows that the proposed image encryption scheme demonstrates high security, efficiency, and robustness, making it effective for image security in various applications.

## 1. Introduction

The importance of image security has grown due to its wide range of applications. Traditional cryptosystems often require significant time to encrypt large color images. As a result, cryptosystems with faster speeds and lower costs are being researched and developed. Recently, chaos-based image cryptosystems have garnered increasing attention for their lightweight nature and high efficiency [1,2]. The initial value sensitivity, ergodicity, and periodic point density of chaotic systems ensure that they are locally random but globally bounded, making their output difficult to predict and meeting the confusion and diffusion requirements of a good cryptosystem. Much research focuses on chaotic systems, attractors, and chaotic sequences to achieve high security for image encryption [3,4,5]. Chaos-based image encryption algorithms can be categorized as either 1D chaos-based or higher-dimensional chaos-based. For instance, 1D chaotic systems with two seed maps are proposed in [6] to create a novel image encryption scheme, transforming the original image into different encrypted images with the same key. Following a similar approach, two different 1D chaotic maps are used in [7] to output sequences for encryption. A cosine-transform-based chaotic system is presented in [8] to scramble and diffuse the image. Although 1D chaotic systems are efficient, they have a small key space and lack complexity, making their orbits predictable, which can lead to security vulnerabilities [9,10,11]. High-dimensional chaotic systems, with more complex behavior and larger key spaces, provide greater security. In [12], a color image is converted into a two-dimensional matrix, which is scrambled using a combined DNA coding operation with a three-dimensional chaotic system and Fisher–Yates scrambling. Mansouri and Wang [13] improved the Arnold system by combining it with a shuffle operation to scramble and diffuse the image. The Lorenz system, a classical chaotic system, has been modified and widely used in chaos-based image encryption and communications [14,15,16]. In [17], the nonlinear term of the general Lorenz system was replaced by the sum of an exponential function and the square of a single variable. This new Lorenz system is then used to generate keys for scrambling image pixels, effectively resisting chosen plaintext attacks. In [18], a coupled chaotic system with complex dynamic behavior is proposed for image encryption, achieving higher security and speed.

DNA coding has been introduced in chaos-based color image encryption [19,20,21,22] to enhance security. These schemes divide the image into three channels and transform them into matrices using DNA coding, then use the chaotic system-generated keys to diffuse the image. Ravichandran et al. proposed a two-level image encryption scheme based on the chaotic map and deoxyribonucleic acid (DNA) [23,24]. In [25], a 4D cat map and elliptic curve ElGamal are used to encrypt color images, resulting in high resistance to known attacks. Numerous other chaos-based color image encryption schemes have also been proposed [26,27], all demonstrating high security and robustness. However, chaos-based encryption schemes often require multiple pixel scans and sorting operations, leading to high computational complexity [28,29,30]. Furthermore, the binarization of chaotic systems introduces degradation, reducing security.

Deep learning is being used for image encryption due to its nonlinear structure, though it is still in its early stages. Ding and Zheng et al. proposed image encryption schemes based on GAN, cycle-GAN, and their variants [31,32]. In GAN-based encryption, a set of encrypted images is used as hidden factors to train the network, which transforms plain images into cipher images. In [32], cycle-GAN is used to disguise plain images with cover images. In [33], plain images are diffused before being passed through the encryption model, where GAN is used as the encryption component. Wang et al. generated cipher images directly, without training a neural network, using scrambled DCT coefficients instead. Another method uses deep learning to generate secret keys [34,35], achieving a high key space. However, convolution operations in deep learning without normalization cause pixel values to exceed 255, and normalization results in float values that cannot be displayed as images. Consequently, deep learning is typically used to generate control parameters for image encryption. In [36], the facial image of a person is employed to extract features using a convolutional neural network (CNN). These features are then used to control the sine logistic modulation map, which generates chaotic matrices for the encryption of CT images. Zhou et al. proposed an image encryption scheme based on a conditional generative adversarial network (CGAN) [37]. The primary image is encoded into two noise-like images, which are then used to generate a speckle pattern and trained with the primary image by the CGAN. Upon receiving the ciphertext of the two noise-like images, they are first decrypted and recombined into the speckle pattern. This speckle pattern is then input into the CGAN to output the corresponding original image. Panwar et al. summarized the latest deep learning-based image encryption methods, analyzing their advantages and possible vulnerabilities to attacks [38]. These studies show that deep learning-based image encryption schemes may be vulnerable to attacks common to deep learning models [38,39], such as hidden factor leakage and network architecture exposure. Additionally, since the secret keys do not correlate with the plaintext image, they may be compromised by chosen plaintext attacks [40,41].

From the above, we can conclude that chaos-based image encryption schemes, when combined with DNA coding or other nonlinear components, enhance security while increasing complexity. Currently, no image encryption scheme employs the VGG network. Deep learning-based image encryption schemes, such as CNN-based encryption and CGAN-based encryption, may be vulnerable to attacks common to deep learning models. To address these security issues, we designed a lightweight VGG (LVGG) neural network based on VGG-16 [42], which offers high efficiency. We then proposed a LVGG-based image encryption scheme that combines the nonlinearity of deep learning models with the randomness of chaotic systems. The LVGG has fewer layers than the classical VGG. Since the VGG network achieves the same receptive field with smaller convolutional kernels and uses fewer parameters than other CNNs, the proposed LVGG can achieve high efficiency in image encryption.

Our contributions are as follows: (i) We propose an LVGG-based key seed generator that takes a plain image as input, where the LVGG with only 7 layers improves the efficiency of key seed generation. We design a novel 4D chaotic system with complex dynamic behavior, based on the Lorenz system, to generate the key seed. This key seed is used as part of the initial values for generating the secret key, correlating the plain image with the encryption process and enhancing resistance to chosen plaintext attacks. (ii) We design a dynamic substitution box (S-box) to scramble the pixels of the image, improving the encryption’s resistance to statistical attacks. (iii) A dynamic SC layer, along with a convolutional addition and modular operation, is dynamically generated for image encryption. The convolutional addition is applied to the image using a convolution kernel of size 1 × 2, followed by modulo 256 calculations, achieving high efficiency in confusion. Finally, the security and robustness of the proposed scheme are analyzed through simulation.

The remainder of this article is organized as follows. In Section 2, we introduce the VGG network and the Lorenz chaotic system. In Section 3, we present the design of the LVGG-based image encryption scheme. This includes the LVGG network, a novel 4D Lorenz-based chaotic system, and the LVGG-chaos-based pseudorandom generator. The dynamic S-box and SC layer, constructed by the secret key, are used to scramble and diffuse the image. Finally, a convolution kernel is designed for further encryption. In Section 4, we discuss the simulation and security analysis of the proposed scheme. Conclusions are drawn in Section 5.

## 2. Preliminaries

In this section, we introduce the notations for the VGG network, the Lorenz system, and the S-box.

### 2.1. The VGG Network

The VGG network shows that the performance of a neural network can be affected by the depth of the network to a certain extent. There are two classical structures of VGG, referred to as VGG16 and VGG19. The only difference between them is the depth of the network. The network structure of VGG networks in [42] is shown in Table 1:

Here, conv1-64 denotes a convolutional layer with a kernel size of 1 × 1, and 64 refers to the number of channels. The other convolutional layers follow the same convention. In Table 1, six different VGG networks are introduced, denoted as A, A-LRN, B, C, D, and E. The differences between these VGG variants lie in the number of channels used in the convolutional layers and the normalization functions applied. The max-pooling layer uses a filter of size 2×2 with a stride of 2. “FC−n” denotes a fully connected layer with n nodes. By using the same size of convolution kernel (3×3) and and max-pooling (2×2), the network structure is simplified, allowing deeper networks to enhance performance. The total number of layers is defined as the sum of the convolutional and fully connected layers.

### 2.2. The Lorenz Chaotic System

The Lorenz system, proposed in 1963 to model weather patterns [43], is defined as follows:

(1) $$\left\{\begin{array}{l} {\dot{x}}_{1}=a\left(x_{2}-x_{1}\right)\\ {\dot{x}}_{2}=bx_{1}-x_{2}-x_{1}x_{3}\\ {\dot{x}}_{3}=x_{1}x_{2}-cx_{3} \end{array}\right.$$

Here, $x_{1}$, $x_{2}$, $x_{3}$ are the state variables. When a=10, b=28, $c=\frac{8}{3}$, the system is chaotic.

### 2.3. Property of the S-Box

The S-box is a nonlinear component in block ciphers that directly determines the security of the encryption algorithm. Generally, the S-box serves as a substitution table for a given input; by looking up the table, one can obtain the corresponding output. It is crucial for resisting linear and differential attacks on block ciphers. The definition of the S-box was first introduced in [44]: an n×m S-box S is a mapping S: ${\left\{0,1\right\}}^{n}\to {\left\{0,1\right\}}^{m}$. S can be represented as $2^{n}$ m-bit numbers, denoted $\left\{r_{0},r_{1},\cdots ,r_{2^{n}-1}\right\}$, where $S\left(x\right)=r_{x}$ for $0\leq x<2^{n}$, and the $r_{i}$ values are the rows of the S-box.

## 3. The Proposed Image Encryption Scheme LVGG-IE

In this section, we design an LVGG neural network to generate a plaintext image-correlated secret key seed, which has higher efficiency than the classical VGG network. This secret key seed is then used as part of the initial values of the proposed chaotic system, with the other two initial values chosen randomly. The pseudorandom sequence generated by the chaotic system serves as the secret key for constructing the substitution box and for image encryption. Additionally, we design an SC layer using the secret key to further confuse the image. Finally, a convolutional layer with a kernel size of 1×2 is applied to the image’s pixel matrix. The details are as follows.

### 3.1. The LVGG-Based Key Seed Generator

To enhance image encryption against chosen plaintext attacks, the plaintext should be fed into the encryption process. We designed an LVGG-based key seed generator that takes a plain image as input, while the LVGG improves the efficiency of key seed generation. The neural network structure of the proposed LVGG is shown below.

**Figure 1:**
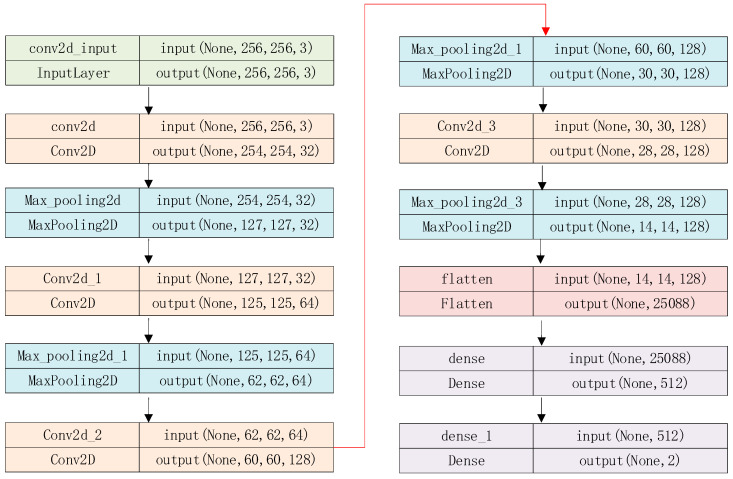
The structure of the proposed LVGG.

In Figure 1, the proposed LVGG neural network contains 7 layers and an input of the 256×256×3 image. The parameter None denotes the batch size, and the output is a vector with size two. The LVGG can classify the image with lower resource consumption and high speed. We trained the network model using a training set of $10^{4}$ images and a test set of $10^{3}$. Both the training set and the test set contain 50% human images and 50% non-human images. All images in these sets were normalized and then resized to 256×256. The number of epochs was set to 10, and the batch size was set to 32. The learning rate was optimized using the RMSprop algorithm, with a value of $10^{-4}$. The network was trained for 10 epochs, achieving an accuracy of 0.788. The proposed LVGG is compared with the VGG 16 and VGG 19 of [42] in Table 2.

From Table 2, we can see that the proposed LVGG requires only 20 s to train the model, which is one-fifteenth of the time needed by VGG 16 and VGG 19. This makes LVGG more efficient for image encryption, especially when the neural network needs to be retrained.

Since the LVGG network uses the softmax function for classification, any image can be input into the LVGG neural network to obtain a vector with two floating point values, ${ks}_{1}$ and ${ks}_{2}$, where ${ks}_{1}\in (0,1)$ and ${ks}_{2}\in (0,1)$ are used as two key seeds. When ${ks}_{1}>{ks}_{2}$, it outputs classification 1; otherwise, it outputs classification 0. Thus, the probability that ${ks}_{1}>{ks}_{2}$ is 78.8% if the input image belongs to a specified type. Although an attacker could correctly guess the type of input image with a probability of 78.8%, they cannot obtain the specific values of ${ks}_{1}$ and ${ks}_{2}$. In other words, the proposed LVGG network can not only utilize certain features of the input image but also resist attacks targeting the neural network. However, the key seed needs to be transmitted to the receiver via a secure channel or public cryptosystem for each image, which improves the complexity of key management.

### 3.2. Pseudorandom Generator Based on LVGG and Chaos

To mitigate the degradation caused by the binarization of the chaotic system, we constructed a four-dimensional (4D) system by adding a new controller to the Lorenz system, which exhibits more complex dynamic behavior. The new 4D system is shown in Formula (2):

(2) $$\left\{\begin{array}{l} \dot{x}=a\left(y-x\right)+eyz\\ \dot{y}=cx+dy-xz-w\\ \dot{z}=xy-bz\\ \dot{w}=hx^{2}+rw \end{array}\right.$$

**Figure 2:**
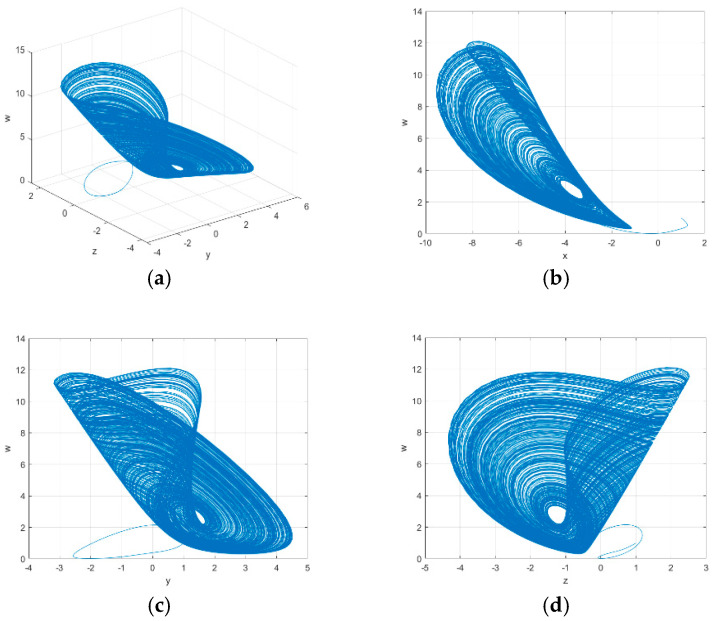
The phase diagram of the proposed chaotic system. (**a**) Phase diagram of the y−z−w plane; (**b**) Phase diagram of the x−w plane; (**c**) Phase diagram of the y−w plane; (**d**) Phase diagram of the z−w plane.

When $a=1,\;b=4,\;c=-1.5,\cdot d\in \{\left[0.95,1.26\right]\cup \left[1.35,3\right]\}$, e=3, h=1 and r=−5, the system becomes chaotic. Its phase diagram is shown in Figure 2:

The Lyapunov exponents are ${LE}_{1}=0.4222$, ${LE}_{2}=0.0698$, ${LE}_{3}=-4.0025$, and ${LE}_{4}=-5.3752$ for d=1.1. The Lyapunov dimension, which represents the complexity of the attractor, can be calculated using Formula (3).

(3) $$D_{Li}=j+\frac{1}{\left|L_{j+1}\right|}\sum_{i=1}^{j}L_{j}$$

where $L_{j}$ is the j-th Lyapunov exponent, and j is the largest index that makes $\sum_{i=1}^{j}L_{j}>0$. The Lyapunov dimension of the proposed scheme is 2.1229. From the above, it can be seen that the proposed 4D chaotic system has a larger chaotic range and more complex dynamic behavior than the Lorenz system, which effectively reduces the degradation of the quantization of the chaotic sequence.

**Figure 3:**
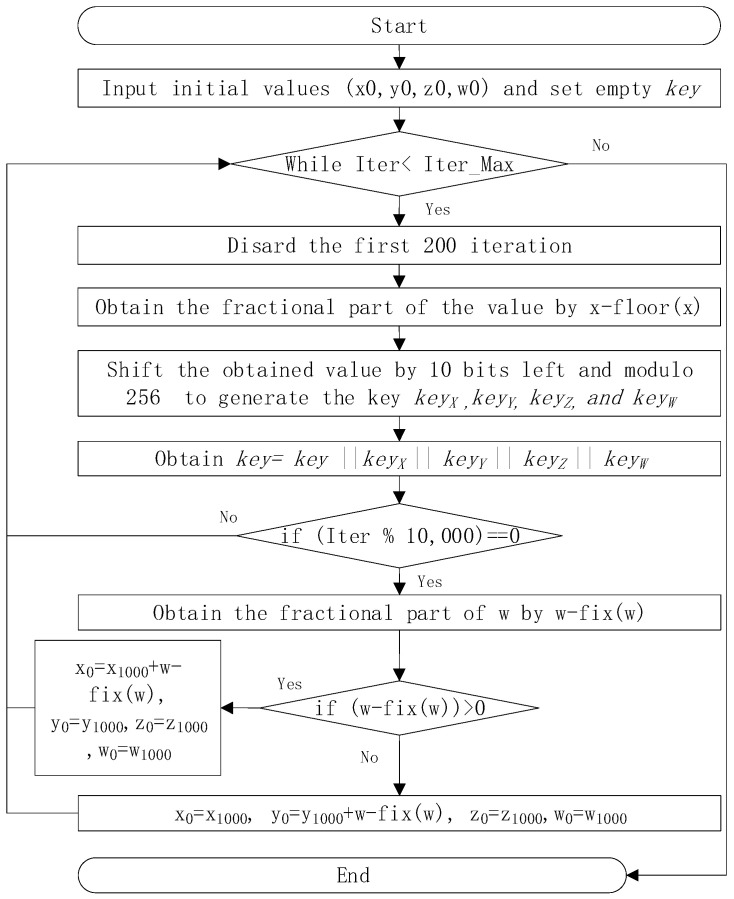
Chaos-based pseudorandom sequence generator.

Additionally, we designed a pseudorandom generator based on the LVGG and chaos, ensuring that the secret key is correlated with the plain image. In the pseudorandom sequence generation process, we use self-perturbation to minimize degeneration. The details are shown in Figure 3.

The pseudorandom sequence generation process is as follows:

Step 1: Input the initial value ($x_{0}$, $y_{0}$, $z_{0}$, $w_{0}$) into the chaotic system, where the output of LVGG ${ks}_{1}$ and ${ks}_{2}$ are added to $x_{0}$ and $y_{0}$, respectively. While $z_{0}$ and $w_{0}$ are chosen randomly. The outputs of the chaotic system are denoted by $x_{i}$, $y_{i}$, $z_{i}$, and $w_{i}$. Discard the values of the first 200 iterations in each 10,000 iterations.

Step 2: Obtain the fractional part of the values $x_{i}$, $y_{i}$, $z_{i}$, and $w_{i}$ by $x_{i}=x_{i}-floor(x_{i})$. Here, floor(x) denotes the largest integer less than or equal to x.

Step 3: Multiply $x_{i}$, $y_{i}$, $z_{i}$, and $w_{i}$ by $2^{10}$ and apply modulo 256 using Formula (4).

(4) $${key}_{x_{i}}=mod(floor(x_{i}\times 10^{10}),256)$$

The binary sequence can be obtained by cascading ${key}_{x_{i}}$, ${key}_{y_{i}}$, ${key}_{z_{i}}$, and ${key}_{w_{i}}$, denoted by $key={key}_{x_{i}}\;||{\;key}_{y_{i}}||\;{key}_{z_{i}}\;||\;{key}_{w_{i}}$.

Step 4: If the iteration is a multiple of 10,000, compute the fractional part of $w_{i}$, denoted as ε. Then, use it to disturb the chaotic system according to Formula (5). Otherwise, continue the iteration.

(5) $$\left\{\begin{array}{c} x_{i}=x_{i}+\varepsilon ,\;\;\;if\;z_{i}>0\\ y_{i}=y_{i}+\varepsilon ,\;\;\;if\;z_{i}<0 \end{array}\right.$$

Since the length of the key generated in each iteration is 32 bits, the length of each of ${key}_{x_{i}}$, ${key}_{y_{i}}$, ${key}_{z_{i}}$, and ${key}_{w_{i}}$ is 8 bits. The key generation process will stop after $({len}_{key}/32)+200$ itertions when $({len}_{key}/32)<10,000$, where ${len}_{key}$ denotes the required key length. It will stop after $\left({len}_{key}/32)+200({len}_{key}\%\;320,000\right)$ itertions when $({len}_{key}/32)\geq 10,000$, where a%b is the remainder when a is divided by b, with $a\in Z^{+}$ and $b\in Z^{+}$.

### 3.3. Design of the Dynamic S-Box

**Figure 4:**
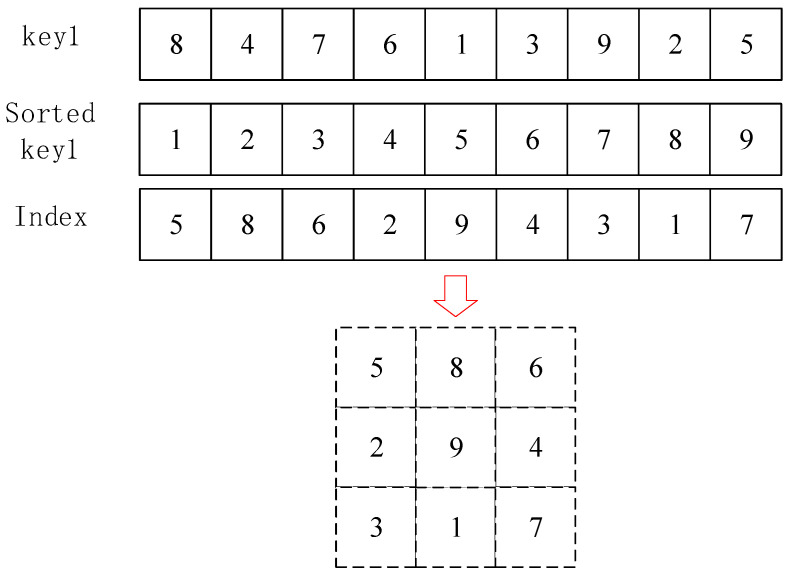
Generation of the dynamic S-box.

To achieve high efficiency, we design a dynamic substitution box (S-box). A sequence s with length 256 is selected from the secret key. This sequence is sorted in ascending order to obtain a new sequence S. The index of the element $S_{i}$ in s is reshaped into a 16×16 matrix, which serves as the dynamic S-box. An example is shown in Figure 4.

To transform the image pixels into cipher image pixels using the S-box, each pixel value of the plain image is divided into the left 4 bits and the right 4 bits. The decimal values derived from these bits represent the row and column numbers of the S-box, respectively.

### 3.4. LVGG-IE-Based Image Encryption Scheme

**Figure 5:**
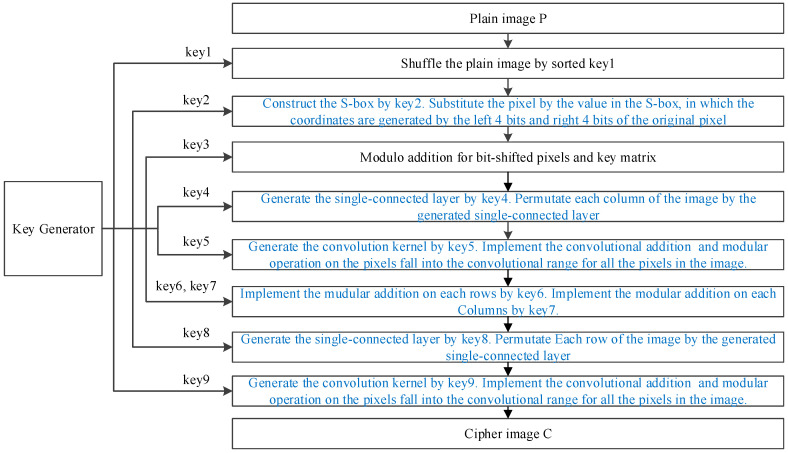
The encryption model.

In this section, we present the proposed image encryption scheme, LVGG-IE, which consists of substitution via the S-box, permutation using the SC layer, convolutional addition, and modular operations. The encryption model is shown in Figure 5.

In this encryption model, the sub-keys are separated from the secret key by key=[key1,key2,key3,key4, key5,key6,key7,key8,key9]. For an image of size row×col, the length of these sub-keys are row×col, 256, row×col, col, 2, col, row, row+1, and 2, respectively. These sub-keys are generated by the pseudorandom generator designed in Section 3.3. Consequently, the secret key for the proposed image encryption scheme consists of the key seed and the other four initial inputs for the proposed chaotic system. Thus, the secret key comprises 6 real numbers, requiring only 384 bits. The details of the image encryption are as below:

Step 1: First, permute the pixels of the plain image using key1. Then, construct the dynamic S-box as described in Figure 4 using key2. Divide each pixel of the image into left 4 bits and right 4 bits, where the decimal values derived from these bits represent the row and column numbers of the S-box, respectively. Next, substitute all the pixels of the image with the corresponding values from the S-box.

**Figure 6:**
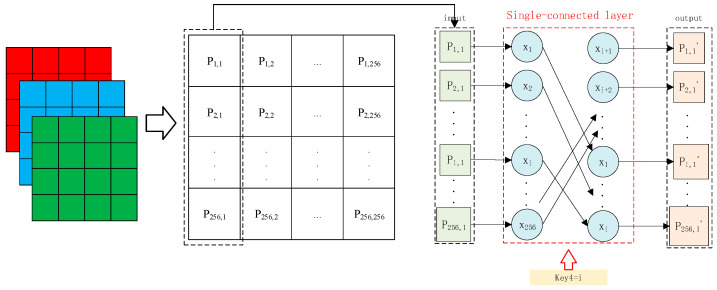
The SC layer-based permutation.

Step 2: Perform modular addition for each bit-shifted pixel of the resulting image and the key matrix generated by key3. Generate the SC layer by key4, and permutate each column of the image using the SC layer, as shown in Figure 6.

Step 3: Generate the convolution kernel Conv by Formula (6):

(6) $$\left\{\begin{array}{c} Conv=\left[1,1\right],\;\;\;if\;key5(1)\;is\;odd\;\;\;\;\;\\ Conv=\left[-1,1\right],\;\;\;if\;key5(1)\;is\;even \end{array}\right.$$

**Figure 7:**
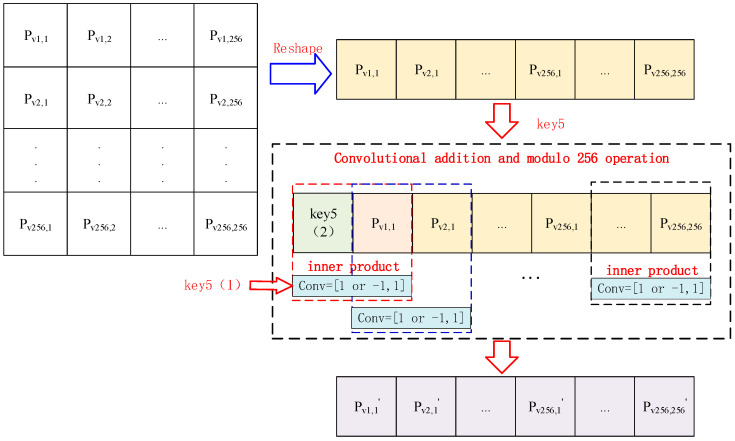
The convolutional addition operation.

Then, transform the n×n image matrix P into one-dimension vectors $P_{v}$ in column order. Perform the convolutional addition operation using the convolution kernel Conv. The pixel value is obtained by applying modulo 256. The details of the convolutional addition are shown in Figure 7.

The different dotted boxes in Figure 7 denote the different convolutional units. The cipher pixel can be calculated by Formula (7):

(7) $$P_{v}^{\prime }\left(i\right)=\left\{\begin{array}{c} mod\left(P_{v}\left(i\right)+\left(2*mod\left(key5(1),2\right)-1\right)*key5(2),\;256\right),\;\;\;if\;i=1\;\\ mod(P_{v}(i)+\left(2*mod\left(key5(1),2\right)-1\right)*P_{v}\left(i-1\right),256),\;\;\;if\;i>1 \end{array}\right.$$

Step 4: Transform the one-dimension vectors $P_{v}$ into the n×n image matrix $P_{c}$. Apply modular addition to each row of $P_{c}$ using Formula (8):

(8) $$\left\{\begin{array}{c} P_{c}\left(i,1:col\right)=mod\left(P_{v}^{\prime }\left(i,1:col\right)+key6\left(1:col\right),256\right),\;\;\;if\;i=1\;\;\;\;\\ P_{c}\left(i,1:col\right)=mod\left(P_{v}^{\prime }(i,1:col)+P_{v}^{\prime }\left(i-1,1:col\right),256\right),\;\;\;if\;i>1 \end{array}\right.$$

Step 5: Apply modular addition to each column of $P_{c}$ using Formula (9).


(9) $$\left\{\begin{array}{l} P_{c}(1:row,i)=mod(P_{c}(1:row,i)+key7(col+1:col+row),256),\;\;\;if\;i=1\\ P_{c}\left(1:row,i\right)=mod\left(P_{c}(1:row,i)+P_{c}(1:row,i-1),256\right),\;\;\;if\;i>1 \end{array}\right.$$

**Figure 8:**
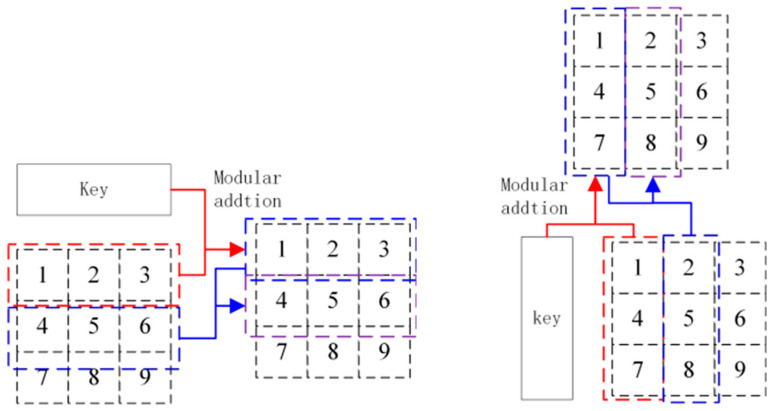
The modular addition on row and column of image.

The process is shown in Figure 8, in which the different dotted boxes denote the different modular addition units:

Step 6: Generate the SC layer by key8 and permutate each row of the image by the SC layer. The operation is similar to that in Figure 6, except that the input is now each row of the image.

Step 7: Generate the convolution kernel Conv by key9(1) and encryption the pixels of the image by convolutional addition and modulo operation, as shown in Figure 7.

The encryption process is detailed in Algorithm 1.

**Algorithm 1.** The image encryption algorithmInput: P,key1, key2, key3, key4, key5, key6, key7, key8, key9 Output: C1:I2←sort(key1)//Obtain the index of the sorted sequence of key12:I←sort(key2)//Obtain the index of the sorted sequence of key23:S←reshape(I,16,16)//Generate the S-box by the index value4:C00←reshape(P,1,row∗col)//Reshape the plain image to one dimension vector5:**for** 
i=1,2,…,row∗col
 **do**
6:  $C01(I2\left(i\right))\leftarrow C00(i)$  //Shuffle the plain image by the index I27:  $C02(I2\left(i\right))\leftarrow Substitute(C01\left(I2\left(i\right)\right),S)$//Substitute the value by S-box8:
**end**
9:

C03←reshape(C02,row,col)

10:

C1←bitshift(C03,3)⊕key3

11for i=1,2,…,col **do** //Permute the image by SC layer12:  $tmp1\leftarrow C1(1:key4\left(i\right),i)$, $tmp2\leftarrow C1(key4\left(i\right)+1:row,i)$13:  C2←[tmp2;tmp1]
14:
**end**
15:C3←reshape(C2,1,row∗col) //Transform the image matrix to one-dimension vector16:C4(1)←mod(C3(1) + 2(mod(key5(1),2)−1)*key5(2),256)//Encrypt image by convolutional addition and modulo 17:**for** i=2,…,col∗row **do**18:  C4(i)←mod(C3(i) + 2(mod(key5(1),2)−1)*C3(i−1),256)19:
**end**
20:C4←reshape(C4,row,col)   //Transform the one-dimension vector to image matrix 21:$C5(1,1:col)\leftarrow mod(C4\left(1,1:col\right)+key6\left(1:col\right),256)$//modular addition on row22:**for** i=2,…,row **do**23:  C5(i,1:col)←mod(C4(i,1:col)+ C4(i−1,1:col),256)24:
**end**
25:$C6(1:row,1)\leftarrow mod(C5\left(1:row,1\right)+key7\left(col+1:col+row\right),256)$  //modular addition on column26:**for** i=2,…,col **do**27:  C7(1:row,i)←mod(C6(1:row,i)+ C6(1:row,i−1),256)28:
**end**
29:for i=1,2,…,row **do** //Permute the image by SC layer30:  $tmp1\leftarrow C7(i,1:key8\left(i\right))$, $tmp2\leftarrow C7(i,key8\left(i\right)+1:col)$31:  C8←[tmp2 tmp1]
32:
**end**
33:C8←reshape(C8,1,row∗col) //Transform the image matrix to one-dimension vector34:C9(1)←mod(C8(1) + 2(mod(key9(1),2)−1)*key9(2),256)//Encrypt image by convolutional addition and modular35:**for** i=2,…,col∗row **do**36:  C(i)←mod(C9(i)+ 2(mod(key9(1),2)−1)*C9(i−1),256)37:
**end**
38:C←reshape(C,row,col) //Transform the one-dimension vector to image matrix39:**return** C

The decryption is the inverse of the encryption. The differences are as follows:

First, the inverse of convolutional addition and modular operations simply requires changing the addition operation to subtraction. The inverse of the SC layer follows the same steps as the decryption process shown in Figure 5. Second, the bit shift XOR operation is modified to $C_{v}\left(i\right)=bitshift(C_{v}\left(i\right),-3)⊕key$. The modular addition of rows or columns is changed to modular subtraction $C(i,1:col)=mod(C\left(i,1:col\right)-C\left(i-1,1:col\right),256)$. Third, the inverse of the S-box is performed by searching for the value of each pixel in the cipher image within the S-box, obtaining its row and column values, and converting them into two 4-bit sequences. These are combined and then converted to a decimal value, which is the decrypted value from the inverse S-box.

## 4. Simulation and Security Analysis

The security of the proposed color image encryption scheme is analyzed in terms of key randomness, key sensitivity, the histogram of the cipher image, the correlation coefficient of adjacent pixels, and information entropy. The scheme’s ability to resist differential attacks, data loss attacks, and noise attacks is also simulated. Since the image “Lena” is not recommended by many journals, we replaced it with the image “Peppers,” which shares similar feature space characteristics [45].

### 4.1. Randomness of the Key

The randomness of the key generated by the LVGG and chaos-based pseudorandom sequence generator in Section 3.2 is tested by NIST SP 800. The initial value for the image “Peppers” is (0.3433926,0.6566074,1.33,0.28). The result is shown in Table 3.

From Table 3, we can see that the key generated by the proposed pseudorandom sequence generator passes all tests, with 10 items passing at a proportion of 100%. This indicates good randomness for encryption. When the same method is applied to the Lorenz system, however, the pseudorandom sequence based on Lorenz fails the SP 800 test. A comparison of the two sequences is shown in Table 3, where most *p*-values for the Lorenz sequence are less than 0.05, which does not meet the SP 800 test requirements. Therefore, the Lorenz-based pseudorandom sequence is insecure for image encryption.

### 4.2. Security and Efficiency Analysis of the Dynamic S-Box and SC Layer

To validate the security of the dynamic S-box, we test its nonlinearity and strict avalanche criterion (SAC) in this section. We also analyze the security of the dynamic SC layer.

#### 4.2.1. Nonlinearity Test

We generate 10,000 S-boxes using the method described in Section 3.3 and calculate their nonlinearity using Formula (10):

(10) $$N_{f}=2^{n-1}(1-2^{-n}\underset{\omega \in \mathrm{GF}(2^{n})}{\max}\left|S_{\left(f\right)}(\omega )\right|)$$

**Figure 9:**
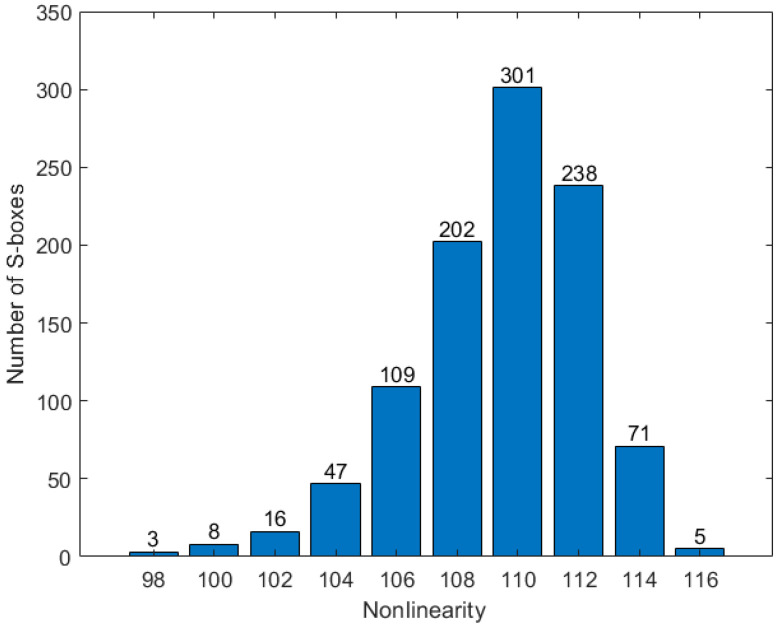
The distribution of the nonlinearity of the S-boxes.

Here, the cyclic spectrum of function f(x) is denoted as $S_{\left(f\right)}\left(\omega \right)=\sum_{\omega \in GF(2^{n})}{\left(-1\right)}^{f(x)⊕x\cdot \omega }$, where x·ω is the dot product of x and ω. The nonlinearity values are shown in Figure 9.

In Figure 9, the number of S-boxes with a nonlinearity greater than 110 exceeds half of the total. Since the nonlinearity of the S-box in AES is 110, the proposed method for generating dynamic S-boxes is secure.

#### 4.2.2. SAC Test

The SAC of the S-box is another significant index for evaluating its security. It is defined such that the S-box is considered secure if its output flips with a probability of 50% when any single input bit is changed. We altered each bit of the input and calculated the flipping probability of each output for 10,000 different S-boxes. The results are shown in Table 4.

In Table 4, we observe that the probability of the S-box output is approximately 0.5 for different inputs. The average deviation from 0.5 is 0.00048, meeting the theoretical SAC deviation requirements.

#### 4.2.3. Security of the Dynamic S-Box and Dynamic SC Layer

The static S-box can be brute-forced using chosen plaintext attacks, where the adversary constructs specific plaintext and obtains the substituted ciphertext through the S-box. In contrast, the dynamic S-box offers higher security since it changes with each encryption. Furthermore, the dynamic S-box proposed in this scheme exhibits high nonlinearity, with the average nonlinearity approaching that of the AES algorithm. It also shows a small deviation from the ideal SAC value. Therefore, the proposed generation method for the S-box ensures high security.

The dynamic SC layer is designed as a shift operation for image encryption in this work. A static SC layer may lead to a statistical attack, in which an adversary could generate different shift results for the plaintext image and deduce the entire SC layer. Based on this guessed SC layer, the adversary could reconstruct the plaintext after the SC shift, rendering subsequent operations ineffective. For example, an attacker might design a plaintext image where the first pixel encrypted by the SC layer is 0, so that the output of the subsequent convolutional addition and modular operation equals the key used in that step. By using a dynamic SC layer, the shift changes with each encryption, making it impossible to guess the SC layer and thereby enhancing security.

#### 4.2.4. Efficiency of Dynamic Generation for S-Box and SC Layer

The dynamic generation of the S-box takes minimal time compared to the entire image encryption scheme. We tested the implementation time on an 11th Gen Intel(R) Core(TM) i7-1165G7 @ 2.80 GHz platform. The results indicate that dynamically generating the S-box requires only 0.0002 s. The dynamic generation of the SC layer requires storage for only col or row bits, as it is merely a rule for bit shifting. Thus, the dynamic method achieves high efficiency.

### 4.3. Encryption Results

**Figure 10:**
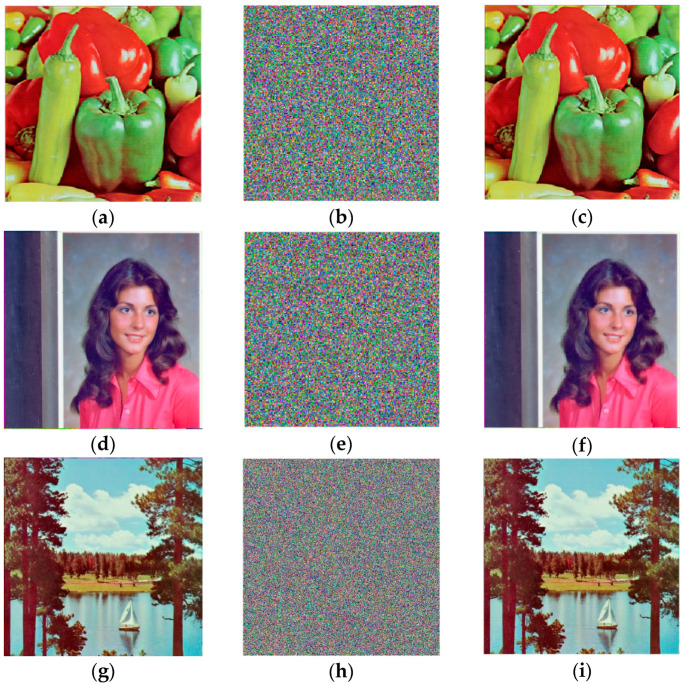
The encryption and decryption results: (**a**) Plaintext image of Peppers; (**b**) Cipher image of Peppers; (**c**) Decrypted Peppers; (**d**) Plaintext image of Female; (**e**) Cipher image of Female; (**f**) Decrypted Female; (**g**) Plaintext image of Lake; (**h**) Cipher image of Lake; (**i**) Decrypted Lake.

In this section, the correctness of the proposed color image encryption scheme is validated. The images “Peppers” (256×256), Lake (512×512), and Female (256×256) are encrypted and decrypted as shown in Figure 10.

The results in Figure 10 show that the cipher images differ from the plaintext and resemble noise. The decrypted images are indistinguishable from the plaintext images, confirming that the proposed scheme can correctly encrypt and decrypt color images.

### 4.4. Key Space and Key Sensitivity

The strength of the image encryption algorithm relies heavily on the robustness of the key. An image encryption algorithm with a key space larger than $2^{128}$ is capable of resisting brute-force attacks. The proposed scheme uses the plaintext to generate the key seed and obtain the key sequence through the proposed 4D chaotic system. The key seed is composed of two floating point numbers with a bit length of 64, giving it a key space of $2^{128}$. The control variables in Figure 2 are x∈[−9,−1], y∈[−3,4.5], z∈[−4,2], and w∈[0,12] when $d\in \{\left[0.95,1.26\right]\cup \left[1.35,3\right]\}$. The key space of the chaotic map is approximately $8\times 7.5\times 6\times 12\times 2.96\times 10^{14\times 5}\approx 10^{74}$ when the key precision is set to 14. Therefore, the total key space is about $10^{112}$. The key space of the proposed scheme is compared with others in Table 5:

In Table 5, the key space of the proposed scheme is larger than that of [20,22], and only slightly smaller than that of [23,25]. This key space is sufficient to resist brute-force attacks.

**Figure 11:**
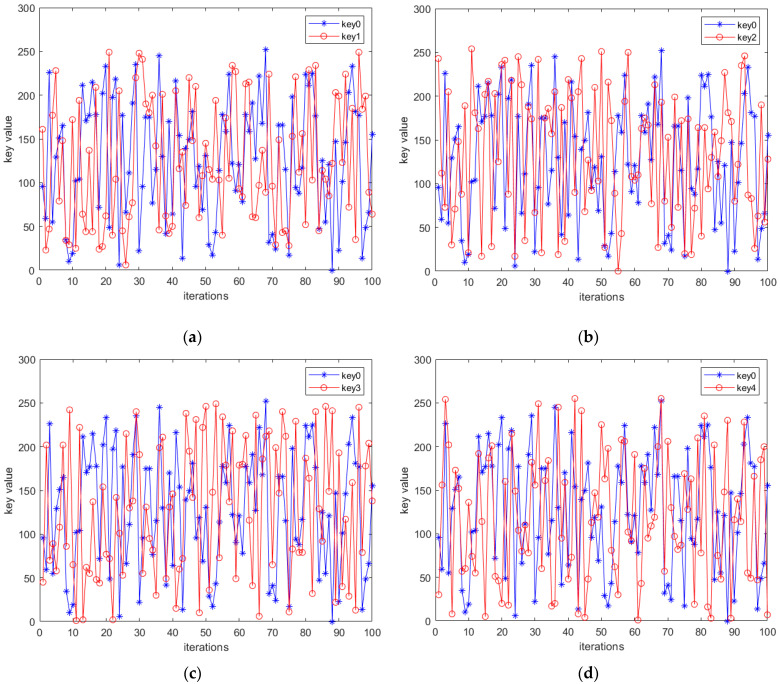
Bit changes in keys with tiny differences in initial value: (**a**) Bit change between $k_{0}$ and $k_{1}$; (**b**) Bit change between $k_{0}$ and $k_{2}$; (**c**) Bit change between $k_{0}$ and $k_{3}$; (**d**) Bit change between $k_{0}$ and $k_{4}$.

Chaotic systems are sensitive to initial inputs, but quantization may reduce this sensitivity. To evaluate the proposed pseudorandom sequence generator’s capacity, we tested the key sensitivity in the encryption and decryption of color images. We generated a key $k_{0}$ using the initial value $X_{0}=(x_{0},y_{0},z_{0},w_{0})=(0.34339260000002,\;0.6566074,\;1.33,\;0.28)$. Then, four different keys $k_{1}$, $k_{2}$, $k_{3}$ and $k_{4}$ are generated by changing $x_{0},y_{0},z_{0}$, and $w_{0}$ by $10^{-14}$, respectively. The sensitivity of these keys to tiny changes is tested. For clarity, the changes are presented in decimal form in Figure 11.

**Figure 12:**
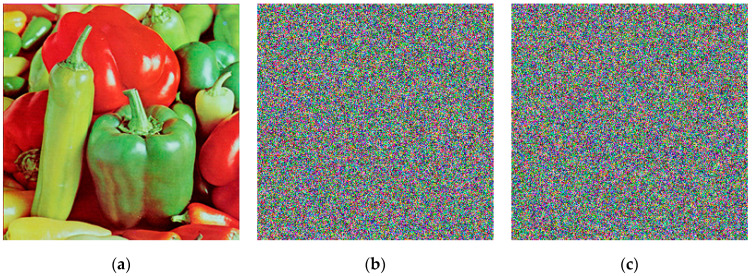

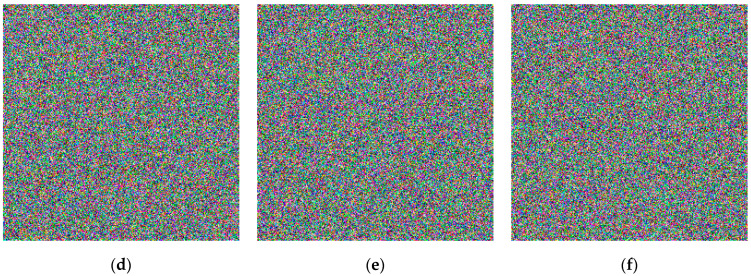
The decryption of the cipher image of Peppers encrypted by $k_{0}$: (**a**) Plaintext image of Peppers; (**b**) Encrypted by $k_{0}$; (**c**) Decrypted by $k_{1}$; (**d**) Decrypted by $k_{2}$; (**e**) Decrypted by $k_{3}$; (**f**) Decrypted by $k_{4}$.

Figure 11 demonstrates that a tiny change of 0.00000000000001 in the initial value results in a complete alteration of the key sequence. This confirms that the key is highly sensitive to changes in the initial value. Additionally, we decrypted the cipher image of “Peppers” (256×256), which had been encrypted using $k_{0}$. The result is shown in Figure 12.

The rate of change between the decrypted images and the plaintext image is shown in Table 6. 

From the data, it is evident that the difference between the decrypted image using a slightly altered key and the plaintext image exceeds 99%. This confirms that the proposed scheme exhibits high key sensitivity.

### 4.5. Histogram Analysis

**Figure 13:**
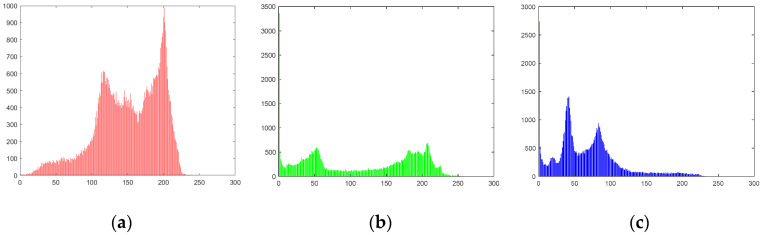

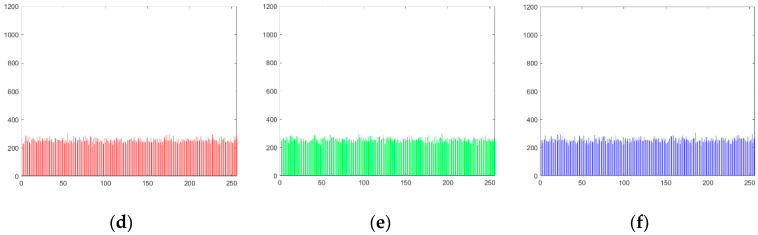
Histogram of the plaintext image Peppers and the cipher image: (**a**) red component of Peppers; (**b**) green component of Peppers; (**c**) blue component of Peppers; (**d**) red component of encrypted Peppers; (**e**) green component of encrypted Peppers; (**f**) blue component of encrypted Peppers.

The histogram describes the distribution of pixel values in an image. A uniform pixel value distribution indicates stronger resistance to statistical attacks. The histograms of the plaintext image “Peppers” (256×256) and its encryption version are shown in Figure 13.

The uniformity of the histogram can be estimated by the variance, which is calculated by Formula (11).

(11) $$var\left(X\right)=\frac{1}{n^{2}}\sum_{i=1}^{n}\sum_{j=1}^{n}\frac{{\left(x_{i}-x_{j}\right)}^{2}}{2}$$

Here, $x_{i}$ and $x_{j}$ represent the number of pixels in the histogram X with gray values i and j, respectively. The parameter n refers to the gray level. Since the variances of the histograms of image in [21] is the smallest, we compare the variances of the histograms of different images for different schemes in Table 7.

Figure 13 shows that the histogram of the cipher image exhibits a uniform distribution, contrasting with the plaintext image’s histogram. From Table 7, we observe that the variances of the histograms of the cipher images encrypted using Algorithm 1 are smaller than those in [21], indicating that the proposed scheme can resist statistical attacks.

### 4.6. Encryption Quality Analysis

In this section, the accuracy of the proposed color image encryption scheme is validated. The quality of encryption is analyzed by examining the closeness of the obtained image to an ideally encrypted image [46]. An ideal encrypted image has a uniform pixel distribution across all intensity levels, which can be assessed using metrics such as deviation from ideality, maximum deviation, and irregular deviation.

#### 4.6.1. Deviation from Ideality

The histogram of an encrypted image generated by a robust encryption scheme should be uniformly distributed. The histogram of the ideal encrypted image $C_{i}$ can be measured by Formula (12), where a small deviation indicates high security:

(12) $$H\left(C_{i}\right)=\left\{\begin{array}{l} \frac{M\times N}{256},0\leq C_{i}\leq 255\\ 0,\;\;\;\;\;\;\;\;\;\;\;\;\;\;\;\;Otherwise \end{array}\right.$$

where M is the number of rows, and N is the number of columns in the image. The deviation from the ideal encrypted image can be calculated using Formula (13):

(13) $$D_{H}=\frac{1}{MN}\sum_{C_{i}=0}^{255}\left|H_{C_{i}}-H_{c}\right|$$

where $H_{c}$ is the histogram of the encypted image.

#### 4.6.2. Maximum Deviation

The maximum deviation (MD) evaluates the difference between the histograms of the cipher and plain images, as calculated by Formula (14). A larger MD represents higher security:

(14) $$MD=\frac{D_{0}+D_{N-1}}{2}+\sum_{i=1}^{N-2}D_{i}$$

where N is the total number of pixel values (for an 8-bit image, $N=2^{8}=256$), and $D_{i}$ is the difference between the i-th histogram of the original and encrypted images.

#### 4.6.3. Irregular Deviation

Since maximum deviation alone may yield inaccurate results in some cases, it cannot be solely relied upon to assess encryption quality. Irregular deviation (ID) measures how close the statistical distribution deviation between the plain and cipher images is to a uniform distribution, as calculated by Formula (15):

(15) $$\left\{\begin{array}{c} ID=\sum_{i=0}^{N-1}{HD}_{i}\\ {HD}_{i}=\left|d_{i}-\mu_{H}\right| \end{array}\right.$$

where $d_{i}$ is the difference between the histogram values of the plain and cipher images, and $\mu_{H}$ is the mean of the histogram values. A higher ID indicates a more uniform pixel distribution.

We calculate the deviation from ideality, maximum deviation, and irregular deviation for the proposed scheme. Additionally, we compare the encryption quality of the proposed scheme using the key seed generated by the LVGG model with that using a random key seed. The results are shown in Table 8.

In Table 8, “Peppers” represents the cipher image encrypted with a key generated from a random key seed, while “Peppers*” represents the cipher image encrypted with a key generated using the key seed from the LVGG network. The results show that the deviation from ideality of the proposed scheme decreases to 0.55, indicating high security. The MD and ID values of the proposed scheme are sufficiently large. Furthermore, the encryption quality of the cipher image using the key seed generated by the LVGG network is better than that using the random key seed. Thus, the proposed image encryption scheme demonstrates high encryption quality.

### 4.7. Correlation Coefficient Between Adjacent Pixels Analysis

**Figure 14:**
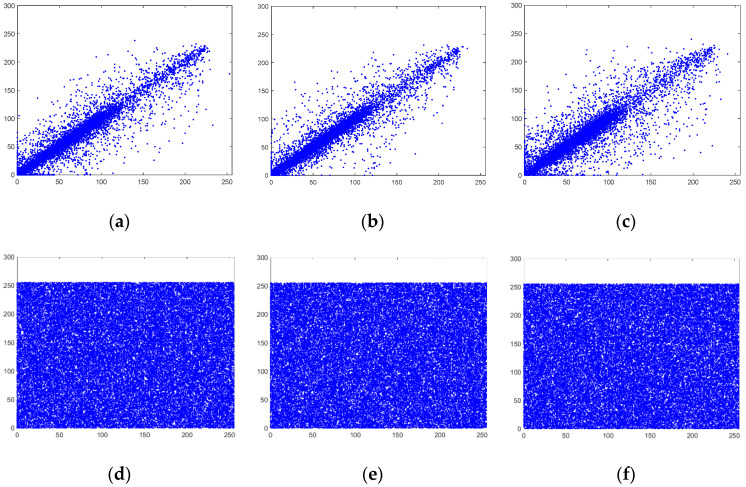
Distribution of adjacent pixels of the blue channel of image Peppers: (**a**) horizontal direction of the plaintext image; (**b**) vertical direction of the plaintext image; (**c**) diagonal direction of the plaintext image; (**d**) horizontal direction of the cipher image; (**e**) vertical direction of the cipher image; (**f**) diagonal direction of the cipher image.

A good image encryption scheme should yield a low correlation coefficient between adjacent pixels in any direction, making it more resistant to statistical attacks. The correlation coefficient between adjacent pixels is calculated using Formula (16).

(16) $$\left\{\begin{array}{l} \bar{x}=\frac{1}{N}\sum_{i=1}^{N}x_{i}\\ D\left(x\right)=\frac{1}{N}\sum_{i=1}^{N}{\left(x_{i}-\bar{x}\right)}^{2}\\ Conv\left(x,y\right)=\frac{1}{N}\sum_{i=1}^{N}\left(x_{i}-\bar{x})(y_{i}-\bar{y}\right)\\ \gamma_{xy}=\frac{Conv\left(x,y\right)}{\sqrt{D\left(x\right)}\sqrt{D\left(y\right)}} \end{array}\right.$$

where N represents the number of chosen adjacent pixels in any direction of the image. In this test, 5000 adjacent pixels were selected to calculate the correlation coefficient between adjacent pixels of both the plaintext and cipher images for “Peppers” (256×256). The result is shown in Figure 14.

The correlation coefficients are summarized in Table 9 and Table 10.

Furthermore, we compare the correlation coefficient between the adjacent pixel of the image Peppers (256×256) with the cipher image Lena (256×256) of other schemes. The lowest correlation coefficient of the three components is chosen to compare with the other schemes. The result is shown in Table 10.

The results show that the correlation coefficients of the encrypted images using our scheme are the lowest, with the minimum coefficient being about one-tenth of that of other schemes. The correlation coefficients of images encrypted using Lorenz-based keys are the highest, indicating that the Lorenz system is inadequate for image encryption due to its poor resistance to differential analysis.

### 4.8. Differential Attack

The number of pixels change rate (NPCR) and unified average changing intensity (UACI) are commonly used to evaluate the ability of an image encryption scheme to withstand differential attacks. If NPCR is close to 100% and UACI is near 33%, the encryption scheme is considered secure against such attacks. Let ${PI}_{1}$ and ${PI}_{2}$ be two plaintext images that differ by only one pixel. There exist that ${PI}_{1}(i,j)\in {PI}_{1}$ and ${PI}_{2}(i,j)\in {PI}_{2}$. ${CI}_{1}$ and ${CI}_{2}$ are generated by encrypting ${PI}_{1}$ and ${PI}_{2}$ with the same key. ${CI}_{1}(i,j)$ and ${CI}_{2}(i,j)$ are any pixel of ${CI}_{1}$ and ${CI}_{2}$ correspondingly, we set $D\left(i,j\right)=1$ if $C_{1}(i,j)\neq C_{2}(i,j)$. Then, the NPCR and UACI can be calculated by the Formula (17):

(17) $$\left\{\begin{array}{c} NPCR=\frac{1}{N\times M}\sum_{i=1}^{N}\sum_{j=1}^{M}D\left(i,j\right)\times 100\%\\ UACI=\frac{1}{N\times M}\sum_{i=1}^{N}\sum_{j=1}^{M}\frac{\left|C_{1}\left(i,j\right)-C_{2}(i,j)\right|}{255}\times 100\% \end{array}\right.$$

where N and M are the numbers of rows and columns in the image matrix. Recently, the theoretical marginal values for NPCR and UACI have been defined in Formula (18).

(18) $$\left\{\begin{array}{c} N_{\alpha }^{*}=\frac{F-\Phi^{-1}(\alpha )\sqrt{\frac{F}{MN}}}{F+1}\\ U_{\alpha }^{*+}=\frac{F+2}{3F+3}+\Phi^{-1}(\frac{\alpha }{2})\sqrt{\frac{\left(F+2)(F^{2}+2F+3\right)}{18{\left(F+1\right)}^{2}MNF}}\\ U_{\alpha }^{*-}=\frac{F+2}{3F+3}-\Phi^{-1}(\frac{\alpha }{2})\sqrt{\frac{\left(F+2)(F^{2}+2F+3\right)}{18{\left(F+1\right)}^{2}MNF}} \end{array}\right.$$

where F represents the largest pixel value supported by the ciphertext image format, and $\Phi^{-1}(\cdot )$ is the inverse cumulative density function of the stand normal distribution N(0,1). The α is the significance level. NPCR results for different images are shown in Table 11.

The UACI of different images is tested in Table 12.

The comparison of NPCR and UACI values for “Peppers” (256×256) and “Lena” (256×256) is presented in Table 13.

The results show that NPCR and UACI values for the proposed scheme fall within the critical value ranges. Additionally, the UACI of the proposed scheme is closer to the critical values compared to other schemes, except for [25]. Thus, the proposed scheme efficiently resists differential attacks. In contrast, the Lorenz-based encryption scheme fails the NPCR test, and its UACI only reaches the $U_{0.001}^{*+}$ critical value, indicating its vulnerability to differential attacks.

Additionally, we use the LVGG network to generate the key seed from the plaintext image, which enhances resistance against chosen plaintext attacks. Attackers could potentially construct specific images, such as an image with only one pixel set to “1” and all other pixels set to “0,” denoted as ${PI}_{1}$. By changing the position of the “1” within the pixel grid or its location in the image, attackers could create multiple plaintext images ${PI}_{i}$. After encrypting all these plaintext images ${PI}_{i}$ to obtain ciphertext image ${CI}_{i}$, attackers could build a map containing ${PI}_{i}$, ${CI}_{i}$ and key. They could then attempt to guess the key by performing subtractions on pairs of ${CI}_{i}$ and analyzing the differences between them. For instance, when $C8\left(1\right)=0$ in Algoruthm 1 for ${CI}_{i}$ and ${CI}_{j}$, the subtraction $\left({CI}_{i}-{CI}_{j}\right)$ can cancel out the convolutional addition and modulo operations from lines 34 to 37 in Algorithm 1. Additionally, key1 could be compromised by collecting more than row×col pairs of tuples $\left({PI}_{i},{CI}_{i}\right)$, since each ${PI}_{i}$ contains only one “1” within the row∗col pixels. The same approach could potentially be applied to obtain key2, and other sub-keys might also be vulnerable to brute-force attacks using this method. On the contrary, when the key is generated from a key seed produced by the LVGG network, each tuple pair $\left({PI}_{i},{CI}_{i}\right)$ corresponds to a different ${key}_{i}$. These vulnerabilities are mitigated because the attacker cannot establish a direct mapping between the tuple pairs and the key.

### 4.9. Information Entropy Analysis

Information entropy reflects the degree of image confusion, which is defined by Formula (19).

(19) $$H\left(m\right)=\sum_{i=1}^{L}p(m_{i})\log_{2}\left(\frac{1}{p\left(m_{i}\right)}\right)$$

where m is the image information, and $p(m_{i})$ is the probability of the gray value $m_{i}$. L is the number of the gray values in image m. The theoretical value of a random image with 256 gary levels is 8. The information entropy of the plain images and the cipher images generated by the proposed scheme is shown in Table 14.

The information entropy of the cipher image “Peppers” (256×256) generated by our scheme is compared with that of the cipher image “Lena” (256×256) from other schemes in Table 15.

Table 14 and Table 15 show that the information entropy of all cipher images generated by our scheme is very close to 8 and is higher than in other schemes, indicating that the proposed scheme is more resistant to statistical attacks. The entropy of the cipher image generated by the Lorenz system is lower, demonstrating less efficiency in resisting statistical attacks.

### 4.10. Robustness Analysis

**Figure 15:**
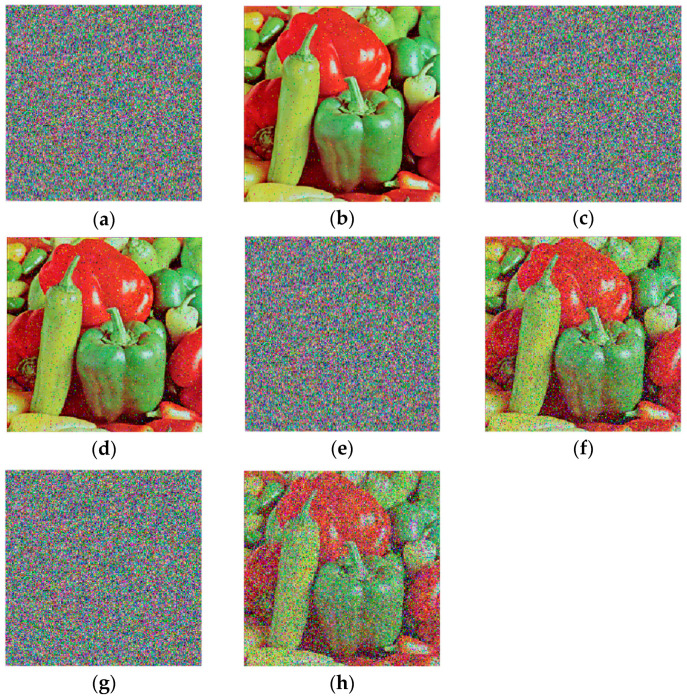
The noised cipher images and their decryptions: (**a**) Noisy cipher image by SPN with density 0.002; (**b**) decryption of (**a**); (**c**) Noisy cipher image by SPN with density 0.005; (**d**) decryption of (**c**); (**e**) Noisy cipher image by SN with variance 0.000002; (**f**) decryption of (**e**); (**g**) Noisy cipher image by GN with variance 0.000001; (**h**) decryption of (**g**).

Since encrypted images may suffer from noise interference or cropping attacks during transmission, robustness against such attacks is essential for an efficient image encryption scheme. We tested the robustness of the image “Peppers” against Gaussian noise (GN), Salt & Pepper noise (SPN), and Speckle noise (SN), as shown in Figure 15.

**Figure 16:**
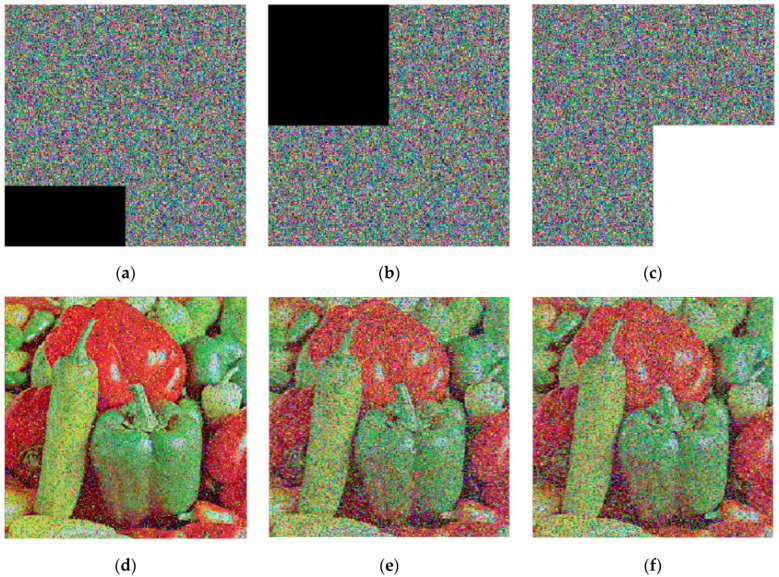
The cropped cipher images and their decryptions: (**a**) cipher image with 1/8 data cropped at the lower-left corner; (**b**) cipher image with 1/4 data cropped at the top-left corner; (**c**) cipher image with 1/4 data cropped at the bottom-right corner; (**d**) decryption of (**a**); (**e**) decryption of (**b**); (**f**) decryption of (**c**).

To evaluate robustness against cropping attacks, different areas of the cipher image of “Peppers” were cropped and then decrypted, as shown in Figure 16.

Figure 13 shows that even cropped cipher images can be partly decrypted. Though some pixels are lost, the image content remains recognizable. Additionally, we used the signal-to-noise ratio (PSNR) to measure resilience to noisy images. A higher PSNR indicates better resilience, and the PSNR is defined in Formula (20).

(20) $$\left\{\begin{array}{l} MSE=\frac{1}{N\times M}\sum_{i=1}^{N}\sum_{j=1}^{M}{\left|P^{\prime }\left(i,j\right)-P(i,j)\right|}^{2}\\ \;\;PSNR=10\times \log_{10}\frac{255\times 255}{MSE} \end{array}\right.$$

where N and M represent the image size, P is the original image, and the noisy cipher image of P is decrypted to $P^{\prime }$. The PSNR for the noisy cipher images is shown in Table 16.

The PSNR values in Table 16 are all above 17 dB for all noise attacks. Even when 25% of the cipher image information is lost, the recovered image remains recognizable, with a PSNR value of around 11 dB. Therefore, the proposed scheme is robust against noise and cropping attacks.

### 4.11. Visual Quality Analysis

The visual quality analysis can be evaluated using MSE and PSNR. The MSE and PSNR values of the plaintext image and the ciphertext image for the proposed scheme are shown in Table 17.

Table 17 shows that the MSE between the plaintext image and the ciphertext image for the proposed scheme is high, and the PSNR is below 10. Therefore, the proposed image encryption scheme demonstrates high security.

### 4.12. Performance Analysis

To validate the efficiency of the proposed scheme, we tested the implementation time and compared it with other schemes, as shown in Table 18.

Table 18 shows that the encryption speed of the proposed image encryption scheme is higher than that of AES and other chaos-based image encryption schemes, making it suitable for image encryption.

From the above, it is clear that the proposed scheme has a high efficiency and capacity to resist statistical, noise, and cropping attacks, making it more secure than other schemes.

## 5. Conclusions

In this work, we proposed a lightweight VGG-based image encryption scheme. By reducing the number of layers and convolution kernel size in VGG, we achieved high efficiency while maintaining a specified input size. The plain image is input into the LVGG network to generate a plaintext-correlated key seed, which serves as part of the input for the proposed novel 4D chaotic system for secret key generation. The dynamic S-box is designed to confuse the pixels of the plain image, while a single-connected layer diffuses the bit-shifted pixels. Finally, the VGG convolution layer is improved to perform convolutional addition and modular operations on the image pixels. In this process, we leverage the advantages of both deep learning and chaotic systems. The proposed scheme’s ability to resist differential, statistical, noise, and cropping attacks is simulated and analyzed. The results show that our image encryption scheme is more secure and robust than state-of-the-art methods.

Although the LVGG-based key seed generation improves resistance against chosen plaintext attacks, it requires an additional secure channel to transmit the key seed for each encryption, which increases the complexity of key management. Future work should focus on improving this aspect to avoid transmitting the key seed for each image. Additionally, the nonlinear properties of deep learning could be further explored to enhance the security and efficiency of image encryption systems.

## Figures and Tables

**Table 1:** The structure of different VGG networks.

A (11 Weight Layers)	A-LRN (11 Weight Layers)	B (13 Weight Layers)	C (16 Weight Layers)	D (16 Weight Layers)	E (19 Weight Layers)
Input (224 × 224 RGB image)					
conv3-64	conv3-64 LRN	conv3-64 conv3-64	conv3-64 conv3-64	conv3-64 conv3-64	conv3-64 conv3-64
maxpool					
conv3-128	conv3-128	conv3-128 conv3-128	conv3-128 conv3-128	conv3-128 conv3-128	conv3-128 conv3-128
maxpool					
conv3-256 conv3-256	conv3-256 conv3-256	conv3-256 conv3-256	conv3-256 conv3-256 conv1-256	conv3-256 conv3-256 conv3-256	conv3-256 conv3-256 conv3-256 conv3-256
maxpool					
conv3-512 conv3-512	conv3-512 conv3-512	conv3-512 conv3-512	conv3-512 conv3-512 conv1-512	conv3-512 conv3-512 conv3-512	conv3-512 conv3-512 conv3-512 conv3-512
maxpool					
conv3-512 conv3-512	conv3-512 conv3-512	conv3-512 conv3-512	conv3-512 conv3-512 conv1-512	conv3-512 conv3-512 conv3-512	conv3-512 conv3-512 conv3-512 conv3-512
maxpool					
FC-4096					
FC-4096					
FC-1000					
soft-max					

**Table 2:** Comparison of the different neural networks.

Networks	Number of Parameters	Time (s)	
		Training	Key Seed Generation
Proposed LVGG	3,453,121	200	0.5
VGG 16 [42]	138,357,544	2890	24.1
VGG 19 [42]	143,667,240	3180	28.9

**Table 3:** The NIST SP 800 test.

Test Item	Proposed Scheme		The Pseudorandom Sequence Based on Lorenz System	
	*p*-Value	Proportion	*p*-Value	Proportion
Frequency	0.23681	0.99	0.006196	0.99
BlockFrequency	0.289667	1	0.028817	0.99
CumulativeSums	0.514124	0.99	0.021999	0.99
Runs	0.304126	1	0.000513	0.99
LongestRun	0.011791	0.98	0.023545	1
Rank	0.637119	1	0.003996	0.99
FFT	0.719747	1	0.162606	0.98
NonOverlappingTemplate	0.999438	1	0.00004	1
OverlappingTemplate	0.401199	0.98	0.030806	1
Universal	0.798139	0.97	0.419021	0.99
ApproximateEntropy	0.494392	1	0.028817	1
RandomExcursions	0.941144	1	0.000691	1
RandomExcursionsVariant	0.848588	1	0.000411	0.9841
Serial	0.383827	1	0.000003	0.98
LinearComplexity	0.23681	1	0.0007	0.99

**Table 4:** SAC of the S-box in the proposed scheme.

Input	$f_{0}$	$f_{1}$	$f_{2}$	$f_{3}$	$f_{4}$	$f_{5}$	$f_{6}$	$f_{7}$
00000001	0.4531	0.4844	0.5000	0.4219	0.5312	0.5469	0.5156	0.5156
00000010	0.4531	0.5781	0.5156	0.5469	0.5312	0.5156	0.4531	0.5469
00000100	0.5312	0.5781	0.5469	0.5312	0.5156	0.5312	0.5000	0.4531
00001000	0.5156	0.5156	0.4375	0.4688	0.4844	0.5156	0.4844	0.5625
00010000	0.4062	0.5469	0.5781	0.4375	0.4844	0.5000	0.5000	0.4844
00100000	0.4375	0.4375	0.4219	0.5312	0.4844	0.6094	0.4531	0.4531
01000000	0.4062	0.5156	0.5781	0.4219	0.5000	0.5312	0.4844	0.5156
10000000	0.5312	0.5156	0.5625	0.4844	0.4531	0.5156	0.4531	0.5156

**Table 5:** Comparison of the key space for different schemes.

Image Encryption Scheme	Key Space
Ravichandran et al. [23]	$10^{126}$
Wu et al. [25]	$10^{117}$
Wu et al. [22]	$10^{90}$
Rehman [20]	$10^{94}$
Ours	$10^{112}$

**Table 6:** Change ratio of the pixel.

Figure	Pixel Change Ratio of Decryption		
	Red	Green	Blue
Peppers	-	-	-
Decrypted by $k_{1}$	0.9962	0.9964	0.9962
Decrypted by $k_{2}$	0.9962	0.9960	0.9964
Decrypted by $k_{3}$	0.9959	0.9960	0.9961
Decrypted by $k_{4}$	0.9959	0.9961	0.9962

**Table 7:** The variances of the histograms of different images.

Image	Scheme	Plaintext Image			Cipher Image		
		Red	Green	Blue	Red	Green	Blue
Peppers	ours	$5.45\times 10^{4}$	$6.43\times 10^{4}$	$10.69\times 10^{4}$	240.18	265.42	248.01
Lena	Ref [21]	$6.24\times 10^{4}$	$2.64\times 10^{4}$	$8.5\times 10^{4}$	247.78	279.62	265.71
Couple	ours	$2.1\times 10^{5}$	$3.37\times 10^{5}$	$2.89\times 10^{5}$	244.54	293.94	263.28
Couple	Ref [21]	$2.1\times 10^{5}$	$3.37\times 10^{5}$	$2.89\times 10^{5}$	284.35	247.37	260.76
Female	ours	$7.9\times 10^{5}$	$8.61\times 10^{5}$	$6.2\times 10^{5}$	310.32	269.23	240.76
Female	Ref [21]	$7.9\times 10^{5}$	$8.61\times 10^{5}$	$6.2\times 10^{5}$	280.64	280.46	230.42
Tree	ours	$8.13\times 10^{4}$	$5.7\times 10^{4}$	$1.29\times 10^{5}$	244.64	232.09	248.51
Tree	Ref [21]	$8.13\times 10^{4}$	$5.7\times 10^{4}$	$1.29\times 10^{5}$	282.81	254.87	225.79

**Table 8:** Comparison of the encryption quality.

Image	Deviation from Ideality			Maximum Deviation			Irregular Deviation		
	Red	Green	Blue	Red	Green	Blue	Red	Green	Blue
Peppers	0.5464	0.5311	0.5242	52,260	36,568	58,576	23,134	35,346	32,500
Peppers*	0.5329	0.5288	0.5204	53,098	37,236	58,819	23,702	35,880	33,414
Couple	0.5537	0.5517	0.5530	86,285	89,183	90,493	30,850	39,334	42,006
Couple*	0.5319	0.5339	0.5290	86,622	89,355	90,698	31,089	39,966	42,152
Female	0.5388	0.5582	0.5555	52,457	51,494	73,061	33,330	30,138	26,816
Female*	0.5116	0.5403	0.5489	52,659	51,763	73,356	33,483	30,538	27,051
Tree	0.5371	0.5584	0.5320	48,239	46,532	62,784	41,678	35,658	39,050
Tree*	0.5328	0.5420	0.5103	48,944	46,932	63,598	41,974	36,013	39,352

**Table 9:** Correlation coefficient between the adjacent pixels.

Figure	Direction	Correlation Coefficients					
		Plaintext Image			Cipher Image		
		Red	Green	Blue	Red	Green	Blue
Peppers	Horizontal	0.94928	0.95959	0.94074	0.00012	0.00328	−0.00355
	Vertical	0.95416	0.96558	0.94979	0.00092	−0.00003	−0.00277
	Diagonal	0.91445	0.9298	0.90273	−0.00286	−0.00118	−0.00332
Couple	Horizontal	0.9556	0.9337	0.92265	0.00122	−0.00553	−0.00040
	Vertical	0.95614	0.95278	0.94711	0.00093	−0.00714	−0.00330
	Diagonal	0.91617	0.9088	0.88896	−0.00169	−0.00453	0.00245
Female	Horizontal	0.97937	0.96848	0.95122	−0.00500	−0.00140	−0.00599
	Vertical	0.98724	0.98146	0.97751	0.00142	0.00379	0.00407
	Diagonal	0.96795	0.95132	0.92774	0.00012	−0.00587	0.00605
Tree	Horizontal	0.95896	0.96869	0.96211	−0.00119	−0.00270	0.00446
	Vertical	0.93627	0.94623	0.9405	−0.00456	−0.00029	−0.00422
	Diagonal	0.91022	0.93092	0.92458	0.00364	−0.00218	−0.00326

**Table 10:** Comparison of the correlation coefficients.

Scheme	Correlation Coefficients				Ranked by Average Value
	Horizontal	Vertical	Diagonal	Average	
Plaintext image (Lena)	0.86939	0.90917	0.85649	0.8787	-
Plaintext image (Peppers)	0.94928	0.95959	0.94074	0.9499	-
Ours (Peppers)	0.00012	0.00092	−0.00286	0.0013	1
Ref. [20]	−0.0073	0.0010	−0.0013	0.0032	4
Ref. [21]	0.0027	0.0033	−0.0035	0.0031	3
Ref. [22]	−0.0084	0.0004	−0.0015	0.0034	5
Ref. [25]	−0.0001	0.0089	0.0091	0.0060	6
Ref. [26]	0.0028	0.0018	0.0036	0.0027	2
Encrypted by Lorenz	0.0019	−0.012	−0.021	0.0116	7

**Table 11:** The NPCR of different images.

Image	NPCR			NPCR Critical Values		
	Red	Green	Blue	$N_{0.05}^{*}$ = 0.995693	$N_{0.01}^{*}=0.995527$	$N_{0.001}^{*}=0.995341$
Peppers	0.995941	0.995895	0.996841	Pass	Pass	Pass
Couple	0.995941	0.995895	0.996841	Pass	Pass	Pass
Female	0.995941	0.996198	0.996487	Pass	Pass	Pass
Tree	0.996216	0.995102	0.996155	Pass	Pass	Pass

**Table 12:** The UACI of different images.

Image	UACI			UACI Critical Values		
	Red	Green	Blue	$U_{0.05}^{*-}$ = 0.332824	$U_{0.01}^{*-}$ = 0.332255	$U_{0.001}^{*-}$ = 0.331594
				$U_{0.05}^{*+}$ = 0.336447	$U_{0.01}^{*+}$ = 0.337016	$U_{0.001}^{*+}$ = 0.337677
Peppers	0.336394	0.335153	0.334672	Pass	Pass	Pass
Couple	0.333639	0.334626	0.335963	Pass	Pass	Pass
Female	0.335716	0.335668	0.336635	Pass	Pass	Pass
Tree	0.333633	0.335124	0.333045	Pass	Pass	Pass

**Table 13:** Comparison of NPCR and UACI.

Scheme	NPCR			UACI		
	Red	Green	Blue	Red	Green	Blue
Ours	0.9959	0.9959	0.9968	0.3364	0.3352	0.3347
Ref. [20]	0.9961	0.9961	0.9961	0.3343	0.3343	0.3342
Ref. [21]	0.9960	0.9961	0.9961	0.3356	0.3345	0.3349
Ref. [22]	0.9961	0.9961	0.9961	0.3346	0.3350	0.3348
Ref. [25]	1	1	1	0.3355	0.3342	0.3344
Ref. [26]	0.9968	0.9966	0.9966	0.3342	0.3342	0.3343
Encrypted by Lorenz	0.986938	0.994246	0.992371	0.332312	0.332861	0.332167

**Table 14:** Information entropy of the plaintext images and the cipher images.

Image	Plaintext Image			Cipher Image		
	Red	Green	Blue	Red	Green	Blue
Peppers	7.3449	7.5607	7.1003	7.9971	7.9973	7.9972
Couple	6.2499	5.9642	5.9309	7.9974	7.9973	7.9974
Female	7.2549	7.2704	6.7825	7.9974	7.9967	7.9970
Tree	6.9207	7.4136	7.2104	7.9967	7.9969	7.9970

**Table 15:** Comparison of information entropy.

Scheme	Information Entropy			Ranked by Average Value
	Red	Green	Blue	
Ours	7.9971	7.9973	7.9972	1
Ref. [20]	-	-	-	-
Ref. [21]	7.9973	7.9969	7.9971	2
Ref. [22]	7.9893	7.9896	7.9903	6
Ref. [25]	7.9912	7.9912	7.9912	5
Ref. [26]	7.9965	7.9963	7.9964	3
Encrypted by Lorenz	7.9923	7.9952	7.9967	4

**Table 16:** The PSNR of the noisy cipher images.

Item	PSNR (dB)		
	Red	Green	Blue
SPN with density 0.002	12.8633	11.2245	11.2165
SPN with density 0.005	25.2144	24.3228	23.7920
SN with variance 0.000002	21.6322	19.7540	19.5796
GN with variance 0.000001	15.9396	14.2369	13.9492
1/8 data cropped at the lower-left corner	15.4233	14.2925	14.1868
1/4 data cropped at the top-left corner	12.5953	11.1434	11.1340
1/4 data cropped at the bottom-right corner	12.6446	11.2594	11.2602

**Table 17:** MSE and PSNR of the plaintext and ciphertext images for the proposed scheme.

Scheme	MSE			PSNR		
	Red	Green	Blue	Red	Green	Blue
Peppers	8032	11,300	11,101	9.0826	7.6001	7.6773
Couple	14,051	15,907	16,173	6.6538	6.1150	6.0430
Female	9399.6	9117.2	6973.1	8.3997	8.5322	9.6965
Tree	8785.2	14,140	9685.3	8.6933	7.5578	8.2697

**Table 18:** Comparison of the implementation time.

Scheme	Encryption Speed	
	Encryption Speed (Mbps)	Decryption Speed (Mbps)
AES	2.6907	2.6907
Wu et al. [22]	3.348	3.348
Chai et al. [21]	1.28	1.28
Ours	3.423	3.569

## Data Availability

Data are contained within the article.
